# Vaccine responses in ageing and chronic viral infection

**DOI:** 10.1093/oxfimm/iqab007

**Published:** 2021-04-19

**Authors:** Chloe Rees-Spear, Laura E McCoy

**Affiliations:** Division of Infection and Immunity, University College London, London, UK

## Abstract

Over the last few decades, changing population demographics have shown that there are a growing number of individuals living past the age of 60. With this expanding older population comes an increase in individuals that are more susceptible to chronic illness and disease. An important part of maintaining health in this population is through prophylactic vaccination, however, there is growing evidence that vaccines may be less effective in the elderly. Furthermore, with the success of anti-viral therapies, chronic infections such as HIV are becoming increasingly prevalent in older populations and present a relatively unstudied population with respect to the efficacy of vaccination. Here we will examine the evidence for age-associated reduction in antibody and cellular responsiveness to a variety of common vaccines and investigate the underlying causes attributed to this phenomenon, such as inflammation and senescence. We will also discuss the impact of chronic viral infections on immune responses in both young and elderly patients, particularly those living with HIV, and how this affects vaccinations in these populations.

## INTRODUCTION

The global population is growing steadily older, with one in six people expected to be over the age of 65 years by 2050 [1]. This expandingdemographic is more susceptible to a variety of illness and disease, and is a major target for public health research [2]. The importance of understanding this group’s increased vulnerability has been particularly exemplified in the recent SARS-CoV-2 pandemic, which has resulted in significantly worse outcomes in elderly individuals [3, 4]. As the body ages, the immune system undergoes a process of immunosenescence, whereby there is a gradual decline towards dysregulation and impaired function. This immunosenescence is linked to a variety of factors, some contradictory, with a general trend towards increased inflammation and cellular dysregulation [5]. This reduced functionality prevents elderly individuals from effectively resolving infections and heightens the importance of prophylactic vaccination in this group. In addition to the increasing population of healthy elderly individuals, there is also a growing population of ageing individuals living with chronic diseases such as obesity and chronic infections. These include infections with viruses that have a latency phase within their life cycle [a classical example being herpesviruses such as human cytomegalovirus (CMV)] and viruses where infection is subclinical (like hepatitis B and C) or viral replication can be suppressed (such as HIV) due to the success of anti-viral therapies [6]. Vaccine coverage for the elderly and those living with chronic conditions is especially critical given their compromised immune systems, but efficacy and uptake of vaccines in these groups (particularly those living with HIV [7]) is variable. Understanding how the combined effect of ageing and chronic illness impact immune responses to vaccination will be critical to improving care for these groups.

The primary purpose of vaccination is to induce a protective immune response against the potential pathogen, this almost always comprises a functional antibody response against a pathogen antigen. For example, the haemagglutinin (HA) protein of influenza or surface antigen of hepatitis B virus (HbSAg). Thus, the protective correlate of most vaccines is a high titre of serum antibodies that can successfully neutralize the vaccine-targeted pathogen. Production of antigen-specific antibodies requires an effective B cell response that is assisted by efficient T cell help and is linked to wider responses to vaccination. Here, we will focus on how age and chronic disease impact that response, and what it means for the success of vaccination in those populations. We will investigate differences in responses to a variety of viral and bacterial vaccines in older and chronically-infected individuals compared to young and healthy individuals, and whether they induce protective titres of functional antibodies in those groups. We will also consider the mechanistic roles of how altered B, T and natural killer (NK) cell functions underly these differences. The involvement of inflammation and specific cytokines in these altered responses will also be discussed. This detailed basis will allow consideration of how chronic disease states are interlinked with immune ageing and reduced vaccine responses. In particular, we will focus on the impact of HIV on vaccine efficacy, which is a pertinent issue for the growing number of people living with HIV above the age of 60 years and are therefore at greater risk due to poor vaccine responses.

## ARE ANTIBODY RESPONSES LOWER IN ELDERLY RECIPIENTS?

Numerous studies have been conducted to investigate the effect of ageing on antibody responses to viral vaccination, particularly against influenza. A 2011 study comparing responses in elderly (70–100 years old) and adult (18–51 years old) recipients of the 2009 seasonal trivalent inactivated influenza vaccine demonstrated that elderly individuals had significantly lower influenza HA-specific antibody titres, as well lower total IgG, 1 week after vaccination. Only 57% (12/21) of elderly volunteers were noted as positive responders (antibody titre increased >4-fold) compared to 90% of adults. The frequency of antigen-specific antibody-secreting cells (ASCs) was also significantly lower in elderly subjects, however, the IgG yield per ASC was similar between groups; suggesting the ability to produce antibodies is not compromised in the elderly [8]. A separate 2011 study examining HA-specific antibody responses to the monovalent H1N1pdm09 influenza vaccine in elderly (66–83 years), adult (46–65 years) and young adult (18–45 years) participants, found that elderly individuals have lower seroconversion rates (60% versus 78% and 94% in adults and young adults, respectively). However, geometric mean HA-antibody titres and seroprotection rates were similar for responders (post-vaccine HA-inhibition titre of >1:128) across all groups. In fact, post-vaccination serum samples from elderly individuals had significantly higher avidity and >5-fold greater HA-antibody binding than the younger cohorts [9]. The authors hypothesize that this improved titre and protection against the H1N1pdm09 vaccine in elderly responders, inverse to what is expected, could be due to the presence of cross-reactive long-term memory B cells induced by previous exposure that are rapidly recruited to produce functional antibodies to H1N1pdm09 [9]. A 2016 analysis of a 4-year study of young adults (16–39 years) and elderly individuals (62–92 years) receiving a seasonal trivalent influenza vaccine found that post-vaccination HA-antibody geometric mean titre (GMT) was roughly twice as high in young subjects compared to elderly. Using linear regression models to predict antibody response post-vaccination demonstrated that pre-vaccination titre and age as independent variables give a predictive power of roughly 30%, indicating that age is a significant factor in determining antibody response. However, the authors also note that this significance decreases with an increasing number of previous vaccinations, with the difference in antibody titre changing from −1.46 to 0.075 over the course of four annual vaccinations [10].

Similar age-related decreases in antibody responses have also been observed following immunization with other viral vaccines. For example, a Phase IV trial comparing primary vaccinations of elderly (61–70 years) and young (18–30) individuals with the inactivated Japanese encephalitis vaccine (JEV) found that the percentage of low and non-responders (antibody titres of <20 on Days 30 and 70 post-vaccination) was significantly higher in the older group (46.7%) versus the younger (13%). The GMT was also significantly lower in the elderly group [11]. A study analysing response to tick-borne encephalitis (TBE) vaccinations in subjects between the ages of 18 and 93, also showed a significant negative correlation between age and antibody titre, regardless of the time elapsed since previous vaccinations. Antibody concentrations were shown to reduce linearly from the time of vaccination, dropping to below protective levels in 5–30% of people over 60 (compared to <2% in people under 60) [12]. A similar effect was observed in a 2009 study that analysed vaccination responses to an inactivated, whole-virus TBE vaccine across four age groups (<30, 50–59, 60–69 and >69 years). TBE-specific IgG and neutralizing antibody concentrations in all groups were significantly lower than the youngest cohort, though no difference was observed between the 50–59 group and the older subjects [13]. These results were corroborated by a later study following old (>50 years) and young (<30 years) subjects vaccinated with inactivated TBE. Significantly lower antibody titres pre- and post-booster, as well as lower neutralizing antibody titres, were detected in the older group [14]. Notably, despite lower titres, there was no significant difference in IgG avidity or in the ratio of neutralizing IgG versus total TBEV-specific IgG, suggesting that the functional quality of antibodies is maintained with age [14]. Furthermore, a 3-year study of responses to the combined hepatitis A/hepatitis B vaccine (Twinrix), in subjects between the ages of 17 and 84, also demonstrated age-related reductions in antibody response. Frequency of seroconversion was significantly decreased with age, with 33% and 63% of those older than 60 protected against hepatitis B and A, respectively (compared with 67% and 92% in those younger than 40) [15].

Together these data suggest that elderly individuals’ antibody responses are lower in response to vaccination against both respiratory and blood-borne viral infections compared to younger cohorts. The magnitude and duration of these differences vary across age groups and studies, but specific antibody titre begins to be significantly reduced from around the age of 50, and much of the protective effect of vaccination is lost with increasing age. However, antibody quality appears to be unaffected, suggesting vaccine hypo-responsiveness in the elderly may be due to defects in cellular induction rather than antibody production.

Age-related changes in antibody response have also been observed following vaccination against bacterially induced disease, most commonly pneumococcal vaccinations, which are recommended in the UK for those over the age of 65 or who are immunocompromised such as individuals with HIV [16]. A 1998 study of 23-valent polysaccharide vaccine-induced antibody activity against *Streptococcus pneumoniae* in elderly (63–103 years) and young (22–46 years) found only a slight drop in titres of vaccine-specific IgG with age. However, the functional opsonophagocytic titres of IgG against all serotypes were significantly lower in the elderly group, and this reduction became even more apparent when this group was stratified by age [17]. This reduced functionality was also observed in a 2008 study of 23-valent polysaccharide vaccination of elderly (>65 years) and young (<45 years) subjects. Specific antibody concentrations were shown to be similar between groups for six out of seven of the serotypes, but opsonophagocytic titres and antibody potency (opsonophagocytic titre divided by antibody concentration) were significantly reduced in older subjects [18]. On the other hand, an analysis of aged responses to tetanus toxoid vaccination observed an almost linear decline in antibody titre after the age of 40, with specific IgG concentration below protective levels when vaccination occurred 6–10 years previously [12]. However, this study did not investigate the functionality of anti-tetanus antibodies in aged subjects.

Contrary to results of most of the viral vaccination studies described above, antibody titre in response to respiratory pneumococcal vaccines in elderly individuals are similar to those of young subjects but functional quality is reduced. Elimination of cross-reactive antibodies in assay measurements has been shown to improve the correlation between antibody titre and functionality and to decrease the significance of any increased antibody titre in elderly subjects >77 years old [19]. This may indicate defects in production of functional antibodies with increasing age, resulting in more non-specific antibodies in elderly individuals. The type of antibodies required by these pathogens may also impact the observed divide between bacterial and viral responses, with opsonophagocytic ability requiring different immune interactions than neutralization. It is interesting to note the difference in titres induced by tetanus toxoid (a protein antigen) versus pneumococcal (a polysaccharide) vaccination. B cell activation against non-conjugated polysaccharide antigens occurs independently of T cell help, whereas the response to protein antigen requires T cell recruitment [20]. Age-associated changes in the T cell compartments may explain the difference in titre between these two vaccines as well as the loss of functionality observed in the pneumococcal response. This is also reflected in the reduced elderly response to viral protein vaccinations, which rely on T cell–B cell interactions to induce the class-switching and affinity maturation that produces functional antibodies. Though the small number of pathogens considered here may limit the ability to extrapolate from these studies, it is likely that the changes in vaccine-induced antibody responses in elderly subjects are due to alterations in aged cellular compartments.

## ARE CELLULAR RESPONSES ALTERED IN ELDERLY RECIPIENTS?

**Figure 1:**
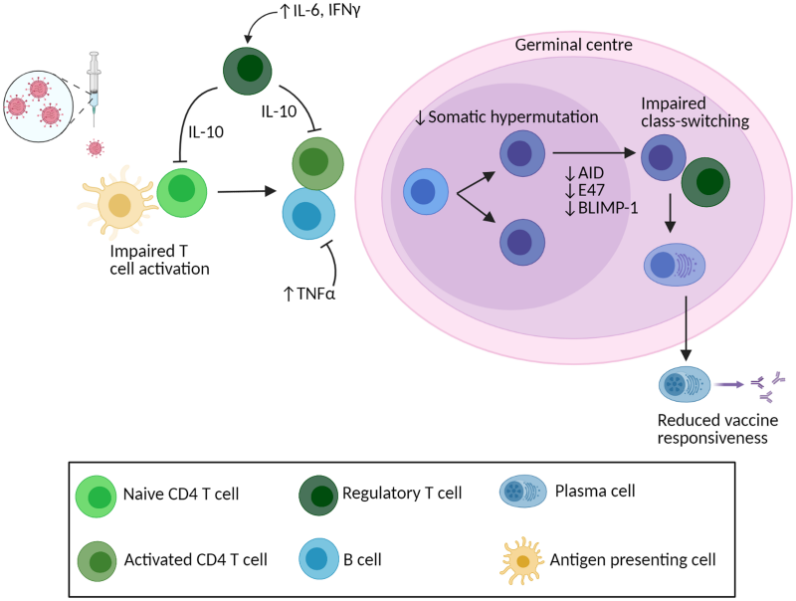
Dysregulated cellular response to vaccination in elderly individuals. Diagram of cellular response to vaccination in elderly individuals. Flat-head arrows indicate actions that inhibit cellular responses. Cell types are as indicated in the legend. Created with BioRender.

There are several potential underlying causes for the lower antibody responses seen in elderly individuals (Fig. 1, Table 1). B cells play a key role in response to vaccination, proliferating into memory and plasma cells that provide long-lasting antibody-mediated protection against subsequent infection. The percentage of naïve B cells present in elderly individuals is significantly lower than in young subjects [11], and a shift towards antigen-experienced memory subsets is a common marker of senescence [5]. The majority of this memory population develops in germinal centres following antigen exposure, where cells undergo somatic hypermutation and class-switching [21]. Primary germinal centre responses have been shown to be severely reduced in aged mice compared to young following immunization with influenza nucleoprotein antigen, resulting in lower antigen-specific IgG [22]. Frequencies of IgG+ memory B cells have also shown to be lower following primary immunization in elderly humans [23, 24]. However, secondary germinal centre responses and levels of memory B cells and long-lived plasma cells are similar in young and aged mice after secondary influenza immunization [22]. A similar result was observed in a human study of elderly (60–80 years) and young (20–31 years) subjects immunized with a TBE vaccine. Following primary vaccination, total CD19+ B cell numbers were 50% lower in older adults, and antigen-specific B cells were also significantly reduced, despite similar levels of naïve B cells prior to vaccination. However, following a 9-month booster vaccination, numbers of antigen-specific memory B cells were similar between young and old despite specific IgG and neutralizing antibody titres continuing to be significantly lower in elderly subjects [25]. The lack of correlation between antibody titre and B cell number is further amplified by the fact that the number of antibodies produced per cell does not appear to be affected by age [8, 25]. This suggests issues with induction and activation of B cells rather than the generation of cellular memory. Alternatively, the disparity could be explained by age-induced effects on the bone marrow plasma cells that produce most serum antibody, but this would be difficult to determine experimentally due to their location. Expression of AID (activation-induced cytidine deaminase, crucial for somatic hypermutation and class-switch recombination) [26], E47 (a transcription factor important for class-switch recombination) and BLIMP-1 (a transcription factor necessary for plasma cell differentiation) [27] is lower in CpG-stimulated B cells taken from elderly individuals, which may hinder proliferation into plasma cells. Furthermore, there is lower expression of genes encoding B-cell receptor components, activation markers, proteins involved in antigen response and germinal centre formation, and chaperones involved in IgM formation in elderly individuals following hepatitis B vaccination (HBV). This reduction in B cell gene expression is correlated with poor HBV response [23]. This raises the concept that the reduced ability of B cells to respond to stimulation, and the resulting changes in gene expression levels associated with antibody production, may be affected by altered interactions with various other cells in the surrounding environment, which will now be discussed.

**Table 1:** Changing immune responses impact antibody titre in response to vaccination

Immune parameter	Impact on antibody titre	Immune status
Expansion of naïve B cells	Increased	Young/healthy
Shift to antigen-experienced B cells	Decreased	Old/chronic infection
Increased naïve CD4+ T cells	Increased	Young/healthy
Increased suppressive CD4+ T cells	Decreased	Old/chronic infection
High IFNg:IL-10 ratio	Increased	Young/healthy
Low IFNg:IL-10 ratio	Decreased	Old/chronic infection
Elevated inflammatory cytokines (TNFa, IL-6)	Decreased	Old/chronic infection
Higher CD4+ T cell count	Increased	Healthy/ART
Suppressed HIV viraemia	Increased	Healthy/ART

Change in T cell profiles also occurs with age and impacts responses to vaccination. CD4+ T cell interaction with B-cell receptors and co-receptors triggers production of various effector molecules that direct proliferation and differentiation of the target B cell, ultimately affecting class-switch recombination, isotype switching and somatic hypermutation to produce antigen-specific antibodies [28]. Populations of these CD4+ T cells are significantly altered in elderly individuals. Naïve (CD45RA+ CCR7+) CD4+ T cells are less frequent, and there is a shift towards central (CD45RA− CCR7+) and effector (CD45RA− CCR7−) memory populations [11]. Following vaccination in elderly individuals, it has been noted that there is an increase in naïve and terminally differentiated CD4+ T cells, which are effector memory cells that re-express CD45RA and lack costimulatory molecules CD27 and CD28, and have been shown to suppress immunoglobulin production in B cells [29]. Moreover, a decrease in effector memory cells has been observed and associated with poor response to vaccination in older subjects [11, 30]. Further age-associated changes are also evident in the percentage of highly suppressive effector regulatory CD4+ T cells (CD25+ Foxp3+), which are increased in elderly but not young individuals [11]. The IL-10- and cell-contact-mediated immunosuppressive functions of these cells are associated with poor responses to JEV and varicella-zoster vaccination [11, 31]. Higher percentage of Tregs prior to vaccination in elderly is also associated with poor antibody production as well as reduced persistence of the response, potentially through PD1 and CTLA4 signalling [31]. In fact, inhibition of mTOR has been correlated with improved CD4+ T cell responses to vaccination through direct and indirect reductions in PD1 signalling (which usually inhibits T cell proliferation and function) with correlated improvements in antibody titre and serologic response [32, 33]. It has been further demonstrated in mice that inhibition of mTOR by rapamycin enhances cross-strain protection against influenza in a CD4+ T cell- and B cell-dependent manner [34]. Moreover, the classical role of CD4+ T cells in antibody generation is that of T helper cell priming in lymph nodes. This is an important stage in germinal centre development and proliferation of these CD4+ helper T cells is reduced in aged individuals. Zheng *et al*. have shown that enhanced priming and proliferation of T helper cells improves germinal centre responses in aged mice, demonstrating the importance of T cell responses in antibody production after vaccination [22]. Intriguingly, their study used immune complexes to improve dendritic cell presentation to T cells. This is linked to an earlier study of aged mice immunized against pneumococcal antigen where macrophage-like accessory cells in the spleen were found to be necessary for an effective response and were significantly reduced in aged individuals [35]. Therefore, these studies suggest that antigen-specific B cell priming by innate immune cells, as well as adequate T cell help and regulatory function, is critical in generating functional antibodies. Moreover, it is indicated that changes in these cell populations in elderly individuals influence the ability of B cells to respond effectively to immunization.

Similarly, although NK cells are important in combating infection, their role in the adaptive response to vaccination is poorly understood (reviewed in Ref. [36]). There is a growing body of evidence that supports direct and indirect regulatory roles for NK cells in T cell priming and activation [37, 38] and regulation of humoral responses [39]. In particular, NK-cell-mediated killing of immature dendritic cells has been demonstrated to improve T cell responses to vaccination in mice [40], and they have been shown to play a role in suppression of Tregs [41]. As with B cells, the population dynamics of these cells change within an individual with age. Elderly individuals accumulate more cytotoxic, mature long-lived CD56^dim^ cells, with a concomitant decline in cytokine-producing, immature CD56^bright^ cells [42]. Older subjects also have more terminally differentiated CD57^+^ cells (a marker of senescence) compared to younger subjects [43, 44]. Vaccination is thought to produce cytokine-induced memory-like (CIML) NK cells (CD56^bright/dim^ CD57^−^ NKG2C^+/−^) through innate or T-cell-derived cytokines, and these cells are primarily found within the less differentiated, immature NK cell population. Activation of these cells via antigen-specific memory CD4+ T cell production of IL-2 results in their proliferation and a resulting increase in IFNγ [36]. The effect of age on NK activity is unclear (reviewed in Ref. [45]), but there is some evidence to suggest that decreased activity of NK cells may be correlated with a lower seroconversion rate and poor vaccine responses in elderly individuals [43, 46]. This correlation could be due to changes in the direct and indirect regulatory interactions between NK and T cell compartments that have knock-on effects in adaptive cellular differentiation. In turn, this could limit the priming and help provided to B cells, resulting in the observed lower seroconversion rates in older individuals.

## A ROLE FOR INFLAMMATION?

These age-related changes in cellular responses to vaccination are linked to concomitant changes in the surrounding cytokine and chemokine milieu [47]. It is well established that, with age, the immune system undergoes a process termed ‘inflammaging’, which is characterized by chronic inflammation that is generally thought to result in poor immune responses [48, 49]. However, the effect of these changes on vaccine responses in the elderly is unclear.

There have been numerous changes documented regarding the altered cytokine responses of elderly subjects following immunization; some of which are directly linked to inflammation, others indirectly. For example, production of both interleukin (IL)-1 and IL-2 have been found to be reduced in elderly mice compared to young mice. Lower levels of pro-inflammatory IL-1 are linked to poor vaccine responses, and its addition to aged splenic accessory cells restores their functionality following immunization [35]. Furthermore, enrichment of IL-1ra (which acts to block IL-1 signalling) in elderly subjects is associated with poor responsiveness (antibody titres <5 mIU ml^−1^) against HBV vaccination [23]. This suggests that, although increased general background inflammation is linked to poor vaccine responsiveness, reduction of individual mediators of inflammation can also lower vaccine responsiveness. In the case of IL-1 this is logical, as this cytokine plays a known role in activation and regulation of both the innate and adaptive immune responses [50]. Meanwhile, IL-2, which plays an important role in stimulating T and B cell growth and maturation [51, 52], has also been found to be reduced in elderly subjects [47]. This reduction has been correlated with the increased frequencies of late-differentiated CD45RA+ CD4+ T cells observed in elderly poor responders to vaccination [30, 31]. More frequent numbers of these suppressive cells, combined with less IL-2-mediated stimulation of B cells, could combine to inhibit antibody production as well as limit the ability of aged immune systems to respond to new antigen. Lower levels of IL-2-secreting CD4+ T cells have also been linked to poor memory B cell induction and reduced antibody titres [25], possibly through the reduced action of IL-2 in BLIMP-1 induction [53] (which, as noted previously, is also decreased in elderly subjects [27]).

Other pro-inflammatory cytokines are also dysregulated with age, both before and after immunization. In particular, levels of TNFα and IL-6 tend to be significantly elevated in elderly individuals following immunization [27]. Elevated TNFα and IL-6 have also been linked to altered antibody isotypes in response to H1N1 influenza vaccination; positively correlating with more IgG3 than IgG1 in serum [54]. The authors hypothesized that, due to the decreased longevity of IgG3 compared to IgG1, this association at least partly accounts for the shortened time span and reduced protection of elderly antibody responses. IL-6 is higher in elderly individuals than younger individuals at any time, though there are mixed results as to whether levels increase post-vaccination in elderly subjects [55]. However, whether there is a post-vaccine increase or not across the board, elevated serum IL-6 is linked to poor antibody responses to influenza vaccination [56]. TNFα is similarly found to be consistently elevated in aged subjects compared to younger individuals, with even higher levels in aged individuals that fail to mount antibody responses [56]. This increase in TNFα has also been positively correlated with poor IgG responses, linked to altered CD4+ T cell frequencies in mice [25]. Interestingly, it has been observed that, despite elevated TNFα in Type 2 diabetes patients, the expected inflammation-associated drop in influenza vaccine response does not occur in old or young subjects [57]. The authors hypothesize that this is due to these individuals’ treatment with metformin, which may prevent the inhibitory action of TNFα on B cell responses [57]. Enrichment of interferon pathways leading to expression of *TNF*, along with *IFNG*, has also been correlated with age and reduced antibody titres [23].

The impact of IFNγ-production on vaccine responses is unclear, with variation in the significance of any changes and levels before and after vaccination in different studies. For example, no statistically significant changes in the frequencies of TBEV-specific IFNγ-producing CD4+ T cells have been found, along with no correlation to changes in antibody titre, in mice [25]. Similarly, no changes in antibody isotype frequencies have been observed with changes in IFNγ levels in response to influenza vaccine [54]. By contrast, additional studies of influenza vaccines demonstrated that, although IFNγ increased following immunization in all subjects, young subjects produced higher levels than the elderly and that this response correlated with better antibody titres [55]. On the other hand, in response to the JEV, no change in IFNγ was detected in elderly but a significant increase was seen in young subjects; and again this increase was correlated with greater antibody titre [11]. To add to the complexity, pre-vaccination IFNγ levels may also play a role in regulating protective cellular and humoral vaccine responses. In elderly subjects that developed influenza, IFNγ levels were 10-fold lower from A/H3N2-stimulated PBMCs, and this was a better correlate of poor protection from the subsequent 2003–04 trivalent influenza vaccine than antibody titre [58]. Contrary to these results, IFNγ-producing CD8+ T cells appear to be more frequent in older individuals following vaccination [59] and, as noted previously, *IFNG* gene pathways have been found to be enriched in elderly subjects with low antibody titres [23]. However, it may be that the level of IFNγ alone is less significant in altering antibody responses than the ratio of IFNγ:IL-10. Memory B cells have been shown to produce elevated IL-10 in elderly compared to young subjects [60]. IL-10 is known to enhance B cell proliferation, class switching and survival, as well as having inhibitory effects on T cell function [61]. IL-10 has also been found to be 3-fold higher, and the ratio of IFNγ:IL-10 significantly lower, in elderly subjects that developed influenza compared to those that did not [58]. Similarly, in response to Japanese encephalitis vaccination, although no change was observed in this ratio in elderly individuals, it was significantly increased in young subjects and correlated with better vaccine responsiveness [11]. The overall effect of IFNγ on antibody response to vaccination is difficult to determine, with conflicting evidence of changes in production pre- and post-vaccination as well as an unclear picture of changes in IFNγ-producing cell types. Elevated IFNγ after vaccination appears to be consistent in young individuals with strong antibody responses, which may be expected given the role of IFNγ in anti-viral responses and improvement of antigen presentation by innate immune cells [62]. It could be that the chronic background inflammation seen in elderly subjects causes the increased production of regulatory IL-10, which subsequently hinders IFNγ-mediated improvement of vaccine responsiveness in those individuals.

In addition to changes in cytokine responses, old age is also associated with altered levels of other inflammatory response markers within the immune system. For example, pathways related to granzyme B production are enriched in elderly subjects, and upregulation of genes involved in acute phase responses and Th2 interferons (processes that inhibit B-cell responses) is correlated with low antibody titres following HBV vaccination in older adults [23]. However, in studies of influenza vaccination, low granzyme B levels and activity following vaccination in elderly subjects have been linked to poor vaccine efficacy, despite protective antibody titres [58, 63]. Other markers of inflammation that are found to be elevated in the elderly and linked poor vaccine responses include C-reactive protein [27], red blood cell counts (with associated increases in free haemoglobin triggering Type 1 interferon responses) [23] and microRNAs that inhibit AID and E47 activity in B cell maturation [64]. All of these responses join together to create a web of inflammatory pathways that result in altered cellular profiles and changes in the efficacy of aged vaccine responses.

## THE SYNERGISTIC EFFECT OF CHRONIC DISEASE

As well as the natural decline of the immune system with age, older individuals are more prone to pro-inflammatory co-morbidities such as cardiovascular disease, atherosclerosis and cancer, among others [65]. The inflammatory nature of these conditions further impacts the reduced vaccine responsiveness observed in older adults. For example, obesity, which has higher prevalence among the elderly and is an increasing health risk in aging populations [66], is known to cause chronic inflammation. This inflammation is linked to exacerbated reductions in influenza-vaccine-specific antibody titres in obese elderly subjects, as well as a further drop in switched memory B cell numbers, B cell activation and IgG production compared to lean elderly subjects [67]. Other chronic diseases, such as hypertension, coronary heart disease and arthritis—all pro-inflammatory conditions—have also been associated with poor seroconversion rates following hepatitis B virus (HBV) vaccination. Only 23% of subjects with chronic disease were found to be seroprotected, compared to 55% of healthy subjects and 33% of subjects over the age of 60 [15].

As well as age-related morbidity, elderly individuals are also more likely to carry chronic infections. A major focus of this review is to compare changes in vaccine responses in elderly subjects and those infected with HIV. Furthermore, there is a high prevalence of HIV+ individuals coinfected with CMV and hepatitis B or C, among other co-infections [68, 69]. However, there is a paucity of data when it comes to vaccination responses in these co-infected groups, let alone aging individuals presenting with co-infections. Therefore, we will first consider chronic viral infections that are common in both older and HIV+ populations, and their impact on vaccine responses. The most common age-associated infection is CMV, which is present in a latent state in 50% of people by the age of 40 with increasing incidence with further age [70] and is one of the most common co-infections found in people living with HIV [68, 71]. Although CMV infection is usually asymptomatic as the herpes virus maintains a latent form for much of the infection period [72], it does cause significant morbidity and mortality in situations of suppressed and compromised immunity such as organ transplant [73] and HIV [68]. It is generally accepted that the high burden of CMV in elderly individuals contributes to their chronic inflammation, and that this negatively impacts their susceptibility and outcome to a range of illnesses as well as the effect of vaccination (reviewed in Ref. [74]). The impact of CMV seropositivity has been studied most in-depth as it relates to influenza vaccination, with mixed results (reviewed in Ref. [75]). Elderly non-responders (with <4-fold increase in specific IgG-titre) to influenza vaccination have been associated with significantly elevated anti-CMV IgG [56]. Moreover, CMV seropositivity has been shown to exacerbate the effects of age-related immunosenescence with further decreases in antibody titre following vaccination compared to young and CMV-negative older subjects [11, 44]. Elevated levels of senescent (CD57+) NK cells [43], with reduced IFNγ production [76], have been demonstrated in CMV+ individuals and linked to poor vaccine responsiveness. Similarly, greater populations of late-differentiated CD4+ and CD8+ T cells (with corresponding reduced levels of naïve T cells) have also been found to be more prevalent in CMV+ elderly subjects and are associated with even lower vaccine-induced titres than those found in CMV- elderly [11, 30]. The accumulation of these senescent cell types in CMV infection, beyond the usual age-associated increases, further elevates the levels of pro-inflammatory secretions that appear to diminish vaccine responsiveness in CMV+ elderly individuals. This CMV-associated inflammation also has knock-on effects on B cell responses, with lower AID activation and lower percentages of switched memory B cells correlated with poor influenza H1N1 vaccine-induced titres [24]. By contrast, some studies have found that CMV status in fact has limited to no effect on influenza vaccine responses in older subjects, with no significant impact on average specific antibody titre [77] but reduced protective efficacy against subsequent challenge [63]. Moreover, some studies have suggested that latent CMV infection can improve responsiveness. Murine CMV has been demonstrated to broaden T cell receptor repertoires, improving antigen recognition in subsequent challenge with *Listeria monocytogenes* [78]. In humans, there is some evidence that CMV seropositivity can be linked to improved antibody responses to influenza vaccines [79], though this was only noted in young individuals. These differences may be due to the inherent variability seen elsewhere in studies of older populations or may be due to variation in responses to influenza vaccination, which are affected by levels of prior exposure as well as the level of heterogeneity between the predicted vaccine strains and actual circulating influenza strains. On the other hand, it has also been noted that, in response to multivalent pneumococcal vaccination, age is correlated with poor vaccine responsiveness to some serotypes but CMV seropositivity has no effect on post-pneumococcal vaccine antibody titre and functionality nor on memory B cell frequencies [80].

Similar, and more consistent, immunosenescent effects have been observed in individuals with other viruses that result in subclinical chronic infection rather than classical latency, such as hepatitis B and C. A meta-analysis of adults with chronic hepatitis C virus (HCV) infections that received recombinant HBV vaccines found the proportion of patients that seroconverted following vaccination to be just 0.17, significantly lower than healthy controls [81]. Further studies have suggested that this reduced efficacy may be due to increased PD-1 expression on CD4+ T cells [82], which has also been suggested as a mechanism for reduced antibody responses in elderly subjects [31]. Studies of chronically-infected HCV patients have also shown similar changes in cell populations as elderly subjects, such as increased proportions of IFNγ+ T cells [83] as well as inhibitory T follicular helper and regulatory T cells [84]. Chronic HBV infection has also been demonstrated to cause accumulation of atypical memory B cells (CD21^−^ CD27^−^) and elevated expression of inhibitory PD-1 receptors, which have been linked to impaired antibody production [85]. These altered and dysregulated cellular profiles may link to poor vaccine responsiveness in infected patients and suggest an early induction of senescence in individuals with chronic infections.

Of course, in an analysis of the impact of chronic infection on responsiveness to vaccination, it must be noted that HIV is exceptional in that this infection, when untreated, results in destruction of T cells that leads to general ablation of the immune system. Although most HIV+ patients are immediately placed on ART, this is not always possible in resource-limited settings and immune restoration after prolonged ART is not complete, as will be discussed later. The success of ART means there is a growing population of HIV+ patients that are increasingly subject to age-associated co-morbidities [6]. Globally, the number of people living with HIV over the age of 50 increased from 8% in 2000 to 16% in 2016 [86], with over half of all HIV cases in the USA within this 50+ age group in 2016 [87] and that number is expected to increase. The immunological changes seen in relatively healthy older adults are even more prevalent in age-matched HIV+ individuals (reviewed in Refs [48, 88]), with a combination of age-associated immunosenescence and HIV-induced immunosuppression generally resulting in an even greater overall reduction in immune response (Table 1).

**Figure 2:**
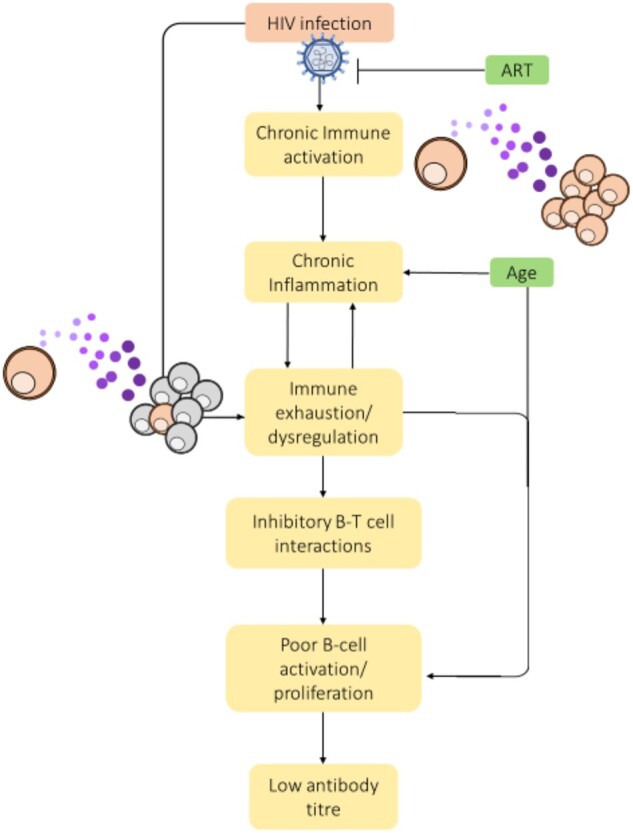
Impact of HIV infection on antibody production in elderly individuals. Schematic of downstream impacts of HIV on the immune response. Flat-head arrows indicate actions that inhibit disease progression. Purple dots indicate the surrounding cytokine milieu that impacts cellular responses.

As is the case in other chronic infections, HIV-infected individuals tend to produce poorer antibody responses following vaccination than healthy adults (Fig. 2). Protective antibody titres have consistently been demonstrated to be lower in HIV+ subjects compared to healthy individuals, with significantly lower numbers of participants classed as vaccine responders following immunization against influenza [89–92], yellow fever [93] and tetanus [94]. The effect of booster vaccinations also appears to be diminished in HIV+ individuals, with no significant increases following a second dose of pneumococcal vaccinations [95, 96] or a third dose of hepatitis A virus (HAV) vaccine (rather than the usual two doses) [97]. However, an additional study of the seven-valent conjugate pneumococcal vaccine found that two doses did improve persistence of the serological response, with 57% versus 69% of subjects maintaining protective titres 5 years after one or two doses, respectively [98]. Further studies have shown that serum antibody responses to a range of vaccinations appear to decline much faster in HIV+ adults. Seroprotective rates from the HBV vaccine have been found to drop to just 40% after 5 years in HIV+ individuals, versus 80% after 10 years in healthy subjects, and an 80–100% reduction in protective hepatitis A titres after 4 years in HIV+ subjects compared to protective titres lasting over 25 years in HIV− subjects [97]. Interestingly, the same study found that titres against the measles, mumps and rubella vaccine (MMR) remain over 95% after 35 years if subjects were vaccinated during childhood before HIV infection, but protective anti-MMR titres in adults drop to 43% after the first year if administered post-HIV infection [97]. As the authors note, this has significant implications for the success of vaccination in children with perinatal HIV infection, particularly in situations where ART availability is limited. The HIV-related studies noted so far have, for the most part, been conducted in young to middle-aged adults, demonstrating the impact HIV has on adult immune systems. Studies that compare these adult responses to those in aged HIV+ subjects (over the age of 55) show that HIV infection has the biggest impact on younger individuals. This is because HIV+ subjects under the age of 40 produce antibody titres similar to older (>55) HIV uninfected subjects [89, 92] and have the lowest seroconversion rates [91] compared to older HIV+ subjects and age-matched HIV uninfected subjects. Thus, it appears that HIV infection induces an immunological state similar to that of uninfected aged individuals, and that greater age in HIV+ subjects does not add greater dysfunction. However, the nature of immune dysfunction arising from HIV infection or age may differ. For example, while studies in HIV-uninfected older individuals point towards reduced antibody titre but similar functionality compared to younger subjects [14], in HIV+ subjects there is evidence that influenza vaccination results in both lower HA-specific IgG and lower neutralisation titres compared to healthy subjects [99]. This indicates that HIV infection not only causes a reduction in antibody titre similar to that seen older individuals, but also impacts the function of vaccine-induced antibody responsiveness and may result in even worse vaccine responses in aged HIV+ subjects than in older HIV−. Overall, these data suggest that HIV infection induces a similar premature immunosenescence to that seen in chronic CMV [24], with younger subjects producing similar or worse antibody responses to vaccination compared to HIV-uninfected older adults. As with age-related immunosenescence, the reduced antibody responses observed in HIV+ patients are due to a number of changes that occur in various cellular compartments as a result of HIV infection.

Consistent with the similarities observed in vaccine-induced antibody responses with greater age and HIV-infection, changes have been observed in B cell populations in HIV+ individuals that are associated with reduced antibody titres. HIV+ individuals have reduced expansion of antigen-specific ASCs post-influenza vaccination, with young HIV+ subjects achieving similar levels as uninfected older subjects, and older HIV+ subjects showing no significant expansion at all [89, 92]. Memory B cells are also known to be impaired in various ways as a result of HIV infection [100]. Poor expansion of memory B cells post-influenza vaccination has been associated with low antibody titre, with HIV+ patients found to have >2.5-fold fewer resting memory B cells before vaccination and significantly less expansion 1 month after [101]. Studies have also shown that, following influenza vaccination, HIV+ individuals have higher frequencies of B cells expressing the inhibitory receptor FcrL4) [90] and markers of exhaustion or senescence (IgD− CD27−) [89] that are associated with low antibody titres. The frequency of PDL1+ (a regulatory receptor) B cells has also been found to be elevated in HIV+ individuals following influenza vaccination, with the most significant increase in HIV+ subjects <40 compared to age-matched healthy controls [89]. Upregulation of PDL1+ on B cells has also been linked to AIDs-related illnesses in the absence of ART and has been shown to be directly induced by HIV virions that have co-opted CD40L from the host cell membrane during viral egress [102]. Together, these data suggest an inhibitory role for PDL1 in B cell activation in HIV patients that impacts antibody production following vaccination. Further changes have also been noted in the frequencies of IL-21R+ B cells. The IL-21/IL-21R signalling axis plays a key role in B cell regulation, development and differentiation [103], and reduced frequencies of IL-21R+ B cells, particularly in late differentiated (CD27− CD10+) populations, has been correlated with low H1N1-specific titres in HIV+ individuals [101]. This decrease in IL-21R-expressing cells post-influenza vaccine also appears to worsen with both age and HIV, with young HIV+ individuals demonstrating similar levels to healthy older subjects and lower frequencies in HIV+ individuals over the age of 55 than in all other groups [92]. In HIV+ individuals with a higher level of blood viraemia, a population of CD21^lo^ tissue-like memory (TLM) B cells have been demonstrated to have elevated expression of a number of inhibitory receptors, such as FcrL4, and to express chemokine receptors that target migration away from lymphoid tissues [104]. These TLM B cells are generally thought to be induced by persistent antigen exposure (similar phenotypes have observed in other chronic diseases such as HCV [105, 106] and HBV [85]) and are considered to be exhausted, with poor ability to expand and respond to stimuli [100]. This would account for poor expansion of B cells and reduced antibody production in HIV+ individuals as they develop an increasingly exhausted B cell population with reduced T cell interaction. These results again paint a picture of early-onset senescence in chronic disease that is either equal to or compounded by the effect of age.

Importantly, several of the changes observed in B cell populations in HIV+ individuals have been linked to changes in T cell frequencies. When left untreated, HIV infection leads to destruction of CD4+ T cell populations, ultimately progressing to AIDS. Despite the advent of suppressive ART normalizing CD4 counts and lessening the level of immunosuppression seen in most modern HIV patients, CD4+ subsets appear to be further functionally impaired as HIV+ subjects age. For instance, the IL-21/IL-21R signalling axis is not only affected by the altered frequency of IL-21R+ B cells. Several studies have also noted decreased production of IL-21 from Tfh cells in HIV+ subjects compared to healthy controls, and that this drop is correlated with both lower frequencies of memory B cells and lower specific antibody titres post-influenza vaccination [90, 92, 107, 108]. Similar to that demonstrated in elderly subjects, cytokine profiles of HIV+ individuals have been shown to be dominated by elevated production of IL-2, IL-17, IL-6 and TNFα following influenza vaccination and are correlated with poor antibody titres [90, 107] as well as low frequencies of resting memory B cells, IL-21-producing Tfh cells and ASCs [92]. Peripheral Tfh (CXCR5+ CD45RA+ CD27+) cells in HIV+ subjects, particularly in those subjects that do not respond to influenza vaccination, have been found to have elevated expression of inhibitory PD1 and decreased expression of ICOS (a B cell co-stimulator), as well as demonstrating reduced ability to stimulate IgG production when co-cultured with B cells [107]. Decreased Tfh production of B cell chemokine ligand CXCL13, which plays a role in B cell organization in the germinal centre [109], has also been observed in HIV+ subjects compared to healthy individuals following influenza vaccination [108]. The combination of decreased CXCL13 produced by peripheral Tfh cells following vaccination, as well as reduced expression of its B cell ligand CXCR5 in HIV+ individuals [110], may result in fewer CD4+ T cell interactions and therefore less B cell differentiation and proliferation that produce the antigen-specific antibody response. This shift in Tfh cell populations towards Th1/Th17 phenotypes with inhibitory co-receptors produces a similar environment of elevated inflammation, decreased cellular responsiveness and poor T cell help as that seen in ageing individuals. Furthermore, frequencies of yellow fever-specific T cells have been shown to be lower in HIV+ individuals 1 year following vaccination compared to healthy subjects, which the authors suggest may impact durability of the response [111]. The early onset immunoscenence that characterizes the antibody, B and T cell response to vaccination in HIV+ subjects appears to be as similarly widespread in chronic infection as in biological ageing.

### Age and time on ART

A point to note when examining the effect of vaccination in adult HIV+ patients is that all the studies mentioned here only include subjects undertaking antiretroviral therapy for a minimum set period prior to study commencement, and all subjects had suppressed viraemia below a certain level. However, the duration of ART and the level of suppression required is not consistent between studies, and some include an additional CD4 count requirement that is also inconsistent, making it difficult to untangle specific effects of these variables and how they may impact vaccine responses. There are also conflicting reports as to how responsive the reconstituted immune system is as a result of ART, despite improved CD4 counts and suppressed viraemia. Continued dysregulation of cellular responses and ongoing inflammation under ART have been reported [112, 113], with some suggestion of persistent HIV replication even with virologic suppression [114–117]. Ongoing immune dysregulation, and particularly the effect of persistent latent infection and inflammation on CD4 T cell populations, would be expected to have noticeable impacts on vaccination responsiveness given the importance of this cell population in mounting effective immune responses. For example, some studies have found that antibody titre following influenza vaccination is proportional to CD4 count at the time of vaccination [94, 118] and that individuals with virologic suppression (<50 RNA copies/ml) and CD4 counts >500 can achieve similar antibody titres to healthy controls regardless of the duration of ART [119]. CD4 count has also been shown to improve responsiveness to pneumococcal vaccination, with significantly higher opsonophagocytic titres in adults with CD4 counts >350 compared to those <350 [120]. Although as no healthy controls were included in that study, the relative impact is hard to determine. However, other studies have found that despite CD4 counts above 500 and ART-mediated virologic suppression, only 56% of HIV+ subjects achieved seroprotective titres following influenza vaccination compared to 80% of healthy controls, with no direct correlation between antibody titre and CD4 count, viral RNA or duration of ART [121]. Similar results have been found following yellow fever vaccination in HIV+ subjects, with higher CD4 counts and suppressed viraemia associated with improved titre but to levels that were still lower than healthy controls [93]. Furthermore, compared to age-matched healthy controls, HIV infection overall appears to have a more significant impact on younger adults (<40) than older (>60) [89, 91, 92, 107], despite all groups being treated with ART. This suggests that despite elderly subjects presumably spending more time on ART, and therefore with a longer period in which to recuperate their immune system [122, 123], their ability to respond to vaccination does not significantly recover after the initial impact of HIV infection. It has also been noted that ART does not necessarily improve the changes in memory B cell populations seen in long-term HIV infection, although studies in children suggest early commencement of therapy may overcome this issue [100]. Similar effects have been observed in other chronic diseases such as HCV, where direct-acting antiviral treatment of chronic infection does not result in reconstitution of functional CD8+ T cells [83]. Furthermore, there is evidence that the impact of HIV infection and long-term toxicity of ART [124] combine to induce early onset of normally age-associated co-morbidities, such as various metabolic disorders and cancers (reviewed in Ref. [125]). For example, as discussed previously, obesity in elderly individuals has been linked to poor vaccine responsiveness [67]. Obesity has also been linked to chronic HIV infection and ART use [125] and is likely to have similar or worse impacts than in relatively healthy older subjects as HIV+ individuals age. There are very few studies that assess the combined effect of age and HIV, along with associated co-morbidities, on the efficacy of vaccination. Given the growing population of individuals under long-term therapy living with HIV into their 50s and beyond, it is clear more investigation into vaccine responsiveness in this group is necessary.

## CONCLUSIONS

**Figure 3:**
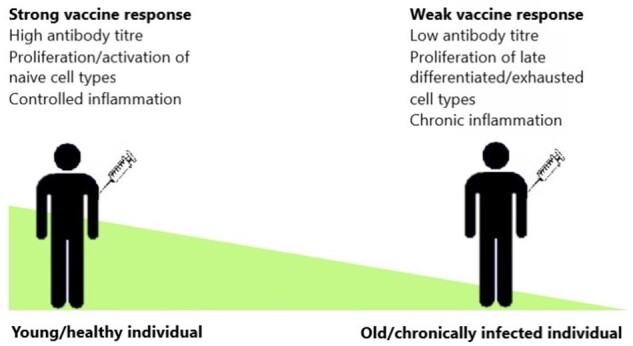
Diagram of impact of age and chronic disease on vaccine response. Elderly and chronically-infected individuals have significantly altered antibody and cellular responses following vaccination compared to those in younger and/or healthy individuals.

Immunoscenence appears to be the cause of consistent reductions in the humoral response and impaired cellular responses to vaccination in both elderly and chronically-infected individuals (Fig. 3). Impaired antibody responses post-vaccination likely arise due to several underlying and interlinked causes. Dysregulation of T cell compartments, due to cell populations skewed away from naïve cell types, as well as elevated inflammatory backgrounds and reduced ratios of IFNγ:IL:10, appear to have significant effect on the induction of B cells. Moreover, potential downstream impacts on germinal centre interactions and maturation of the B cell response further dampens vaccine-induced immunity. There is also significant evidence to suggest that chronic viral infections such as HIV cause lasting immune dysfunction that appears to be only partially recoverable with current therapeutics. Furthermore, it is evident that more research is required to investigate the combined impact of age and chronic infection on vaccine responsiveness in order to effectively protect the growing population of ageing HIV+ individuals. Early analysis of the impact of HIV on the outcome SARS-CoV-2 infection during the recent pandemic suggests that although infection rates appear to be similar between HIV+ and HIV− individuals [126], there is evidence to suggest HIV infection increases the risk of mortality from COVID-19 [127] though this may be due to higher frequencies of comorbidities within this group [128]. Some HIV+ individuals presenting with COVID-19 have been shown to experience elevated inflammatory markers and severe lymphocytopenia despite ART [129], and preliminary data suggests the skewed CD4:CD8 ratio seen in some patients after immune reconstitution negatively impacts T cell responsiveness [130]. However, so far there is limited data dissecting the impact of co-infection. Elderly people have been shown to be particularly vulnerable to SARS-CoV-2 infection and to present with worse disease [131]. Data thus far suggest that both neutralizing antibody titre and T cell responsiveness are important indicators of disease severity [132–134] and that hyperinflammation contributes significantly to COVID-19 pathology [135, 136]. As HIV+ individuals present a similar dysfunctional cellular responsiveness and inflammatory background to older individuals, they may also be particularly vulnerable to circulating virus. Results from Phase 1/2 trials of both the current mRNA and recombinant SARS-CoV-2 protein vaccines suggest that while strong antibody and T cell responses are induced in individuals under 55 [137–139], this is diminished in healthy subjects over the age of 65, though this difference appears to be reduced after administration of a booster in human [140, 141] and mouse [142] studies. Given these preliminary results, investigation into the response of HIV+ individuals to these vaccines will be critical. If mRNA vaccines can induce similar responses to healthy subjects in aged and immunocompromised individuals after a second dose, they could play a crucial role in improving protection in these groups. Though, given the data indicating the quality of immune reconstitution on responsiveness to SARS-CoV-2 in treated HIV+ individuals [130], assessment of CD4: CD8 ratios and levels of viraemia should be considered in determining vaccine scheduling and efficacy. Most studies to date have analysed altered responses to influenza vaccination, however, the variability in exposure, vaccine antigens and circulating seasonal strains make comparisons and extrapolations from these studies difficult. The impact of prolonged ART on these individuals’ ability to respond to vaccination also needs further study. While vaccinations in these immunocompromised populations are an important prophylactic public health measure, research is needed to improve the strength and longevity of vaccine responses in these subjects.

## FUNDING

L.E.M. is supported by an MRC Career Development Award MR/R008698/1 and receives funding from the European Research Council (ERC) under the European Union’s Horizon 2020 research and innovation programme (Grant Agreement No. 757601).

## CONFLICT OF INTEREST STATEMENT

None declared.

## DATA AVAILABILITY STATEMENT

No new data were generated or analysed in support of this work.
